# A New Variant of “Sarcoid Cluster Sign”….But in Tuberculosis

**DOI:** 10.5334/jbr-btr.827

**Published:** 2015-09-15

**Authors:** L. Cardinale, C. Saviolo, G. Cortese

**Affiliations:** 1 Department of Radiology, San Luigi Gonzaga Hospital, Orbassano (Torino), Italy; 2 Department of Radiology, Ordine Mauriziano – Umberto I Hospital, Torino, Italy; 3 Department of Radiology, Maria Vittoria Hospital, Torino, Italy

A 29-year-old Caucasian woman on post-partum was affected by fever, right lower quadrant abdominal pain, irregular alvus and lung micronodules at chest X-ray. Thoracic and abdominal MDCT imaging investigations accompanied by microbiological tests of bronchial aspirate, histology on the surgical bowel specimen resulted positive for pulmonary and intestinal tuberculosis (TB). Volumetric high-resolution computed tomography (HRCT) demonstrated a very interesting pattern about nodules distribution called “Sarcoid cluster sign” (Fig. A). Coronal (Fig. B) and sagittal (Fig. C) Maximum Intensity Projection (MIP) images (18-mm-thick slab) demonstrate the micronodules clusters distribution prevalent in the upper lobes.

**Figures A–C:**
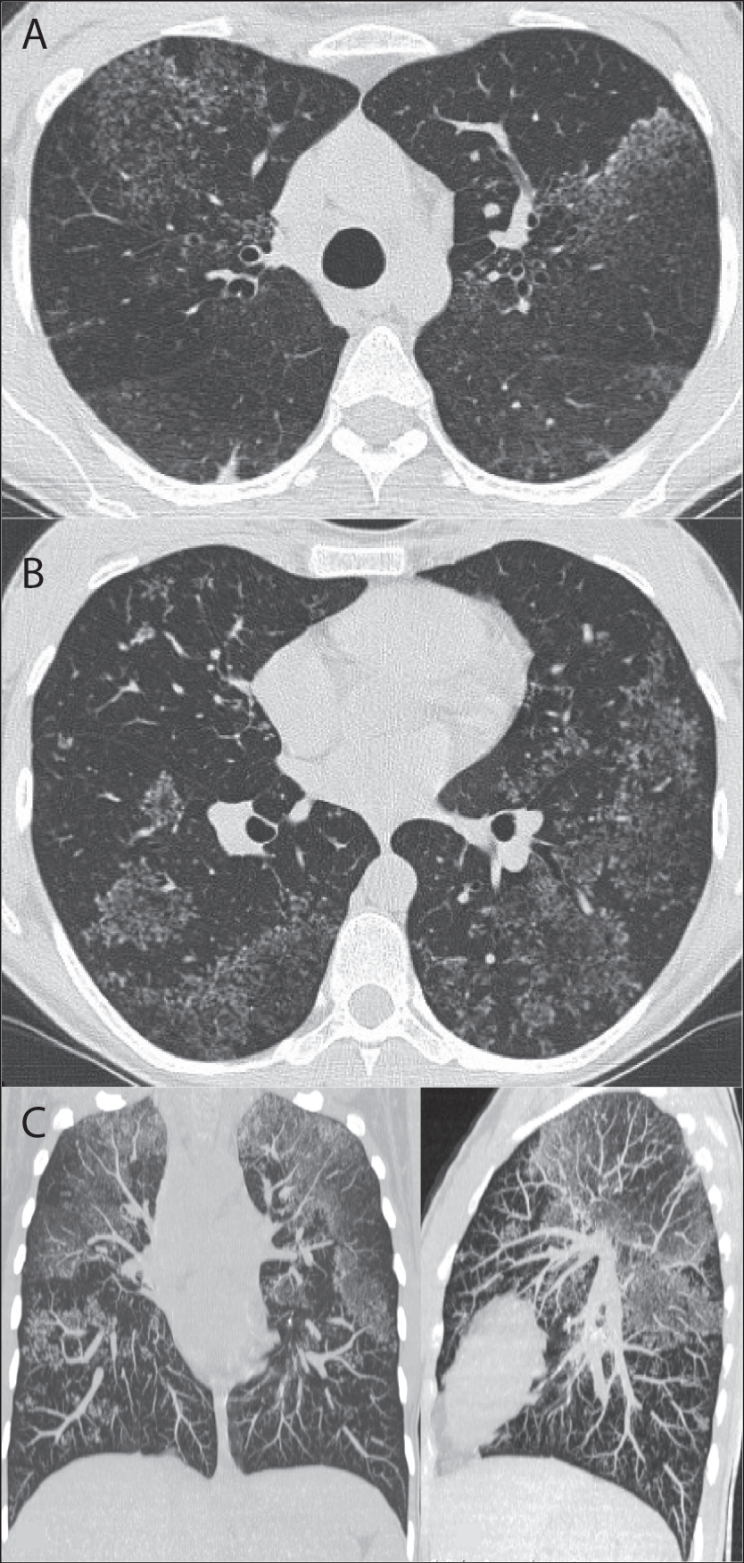


## Comment

This sign was described at the beginning by Ortega et al [1] as a “new HRCT sign, observed in cases of Sarcoidosis”. Marchiori et al [2] described similar findings in a patient with proven pulmonary tuberculosis, concluding that this sign may be seen in both pulmonary Sarcoidosis and pulmonary Tuberculosis. The “sarcoid cluster” correspond to rounded, oval or “tree in bud” clusters of multiple small nodules in the pulmonary parenchyma that are close to each others but not confluent. In our case are also present some areas where these nodules are confluent producing a ground glass appearance. These tiny nodules represent noncaseating granulomas with and without coalescence. The definition of “sarcoid cluster” sign must be extended to confluent micro nodules producing a ground glass appearance.

## Competing Interests

The authors declare that they have no competing interests.
